# Cardiovascular magnetic resonance feature tracking in small animals – a preliminary study on reproducibility and sample size calculation

**DOI:** 10.1186/s12880-017-0223-7

**Published:** 2017-08-23

**Authors:** Tomas Lapinskas, Jana Grune, Seyedeh Mahsa Zamani, Sarah Jeuthe, Daniel Messroghli, Rolf Gebker, Heike Meyborg, Ulrich Kintscher, Remigijus Zaliunas, Burkert Pieske, Philipp Stawowy, Sebastian Kelle

**Affiliations:** 1 Department of Cardiology, Medical Academy, Lithuanian University of Health Sciences, Eiveniu Street 2, LT-50161 Kaunas, Lithuania; 2 Department of Internal Medicine / Cardiology, Deutsches Herzzentrum Berlin, Augustenburger Platz 1, 13353 Berlin, Germany; 3 Center for Cardiovascular Research, Institute of Pharmacology, Charité-Universitätsmedizin Berlin, Berlin, Germany; 4 Department of Internal Medicine / Cardiology, Charité-Universitätsmedizin Berlin, Berlin, Germany; 5 DZHK (German Centre for Cardiovascular Research), Partner Site Berlin, Berlin, Germany

**Keywords:** Cardiovascular magnetic resonance, Feature tracking, Myocardial strain, Small animal model, Reproducibility

## Abstract

**Background:**

Cardiovascular magnetic resonance feature tracking (CMR-FT) is a novel tissue tracking technique developed for noninvasive assessment of myocardial motion and deformation. This preliminary study aimed to evaluate the observer’s reproducibility of CMR-FT in a small animal (mouse) model and define sample size calculation for future trials.

**Methods:**

Six C57BL/6 J mice were selected from the ongoing experimental mouse model onsite and underwent CMR with a 3 Tesla small animal MRI scanner. Myocardial deformation was analyzed using dedicated software (TomTec, Germany) by two observers. Left ventricular (LV) longitudinal, circumferential and radial strain (Ell_LAX_, Ecc_SAX_ and Err_SAX_) were calculated. To assess intra-observer agreement data analysis was repeated after 4 weeks. The sample size required to detect a relative change in strain was calculated.

**Results:**

In general, Ecc_SAX_ and Ell_LAX_ demonstrated highest inter-observer reproducibility (ICC 0.79 (0.46–0.91) and 0.73 (0.56–0.83) Ecc_SAX_ and Ell_LAX_ respectively). In contrast, at the intra-observer level Ell_LAX_ was more reproducible than Ecc_SAX_ (ICC 0.83 (0.73–0.90) and 0.74 (0.49–0.87) Ell_LAX_ and Ecc_SAX_ respectively). The reproducibility of Err_SAX_ was weak at both observer levels. Preliminary sample size calculation showed that a small study sample (e.g. ten animals to detect a relative 10% change in Ecc_SAX_) could be sufficient to detect changes if parameter variability is low.

**Conclusions:**

This pilot study demonstrates good to excellent inter- and intra-observer reproducibility of CMR-FT technique in small animal model. The most reproducible measures are global circumferential and global longitudinal strain, whereas reproducibility of radial strain is weak. Furthermore, sample size calculation demonstrates that a small number of animals could be sufficient for future trials.

## Background

Clinical decision making mostly relies on a quantitative assessment of cardiac structure and function with left ventricular (LV) mass, volumes and ejection fraction (EF) being as critical parameters in many more situations than just selection of appropriate treatment strategy or prediction of cardiac outcomes [1]. However, all these parameters as global quantitative measures have some important limitations [2].

The superior measure for the assessment of global and regional myocardial function is myocardial strain which represents the percentage change in dimension from the resting phase to one achieved of a force or stress [3]. Majority of imaging techniques for myocardial deformation rely on identification and tracking of specific anatomical features. The cardiovascular magnetic resonance (CMR) tissue tracking approach is comparable with speckle tracking echocardiography where myocardium typically has a speckled appearance. In general, a tissue tracking method begins by identifying a relatively small window on one image and searching for the most comparable image pattern in a window of the same size in the subsequent frame [4]. Due to the lack of intramyocardial landmarks and excellent contrast between blood pool and myocardial tissue, CMR feature tracking (CMR-FT) technique focuses on endocardial and epicardial contouring [5].

Measurement of strain using CMR imaging became possible after the introduction of myocardial tagging techniques in which created tagging patterns provide information about myocardial motion during the entire cardiac cycle [6, 7]. However, the need for additional image acquisition and time consuming post-processing makes this technique less attractive.

CMR-FT technique provides quantitative information about myocardial deformation using conventional balanced steady state free precession (bSSFP) cine images and can be used in the assessment of the myocardial mechanics of all cardiac chambers [8–10] and in various clinical scenarios [11–13]. The CMR-FT algorithm is based on optical flow technology and was commercially introduced by several vendors. Interestingly, new calculation algorithms such as non-rigid, elastic image registration has been validated recently and became available in real-life setting [14].

CMR-FT has been validated against myocardial tagging technique for the assessment of regional myocardial motion in humans [15, 16]. Furthermore, recent studies have shown that myocardial deformation parameters may predict future events [17–19] or anticipate response to treatment [20, 21]. Thereby, regional myocardial deformation parameters became a promising new biomarker to detect subtle changes in myocardial motion.

Current literature reports excellent inter- and intra-observer agreement [22–24] and high inter-study reproducibility [25] of quantitative assessment of myocardial mechanics using CMR-FT. Importantly, derived myocardial deformation parameters are similar and highly reproducible in subjects examined at different field strength MRI scanners [26]. However, there are no studies assessing reproducibility of myocardial deformation parameters in small animals.

Accordingly, we performed this preliminary study to evaluate inter- and intra-observer reproducibility of CMR-FT derived strain measurements in a small animal (mouse) model. Also, we calculated the necessary study sample size to define the number of animals required for future studies.

## Methods

Six C57BL/6 J male, mice were randomly selected from the ongoing experimental mouse model onsite. Animals used in this study were maintained in accordance with Guide for the Care and Use of Laboratory Animals published by the US National Institutes of Health. The study was approved by the local authorities (G0099/14).

### Cardiac magnetic resonance

All CMR measurements were performed on a 3 Tesla small animal MRI system (MR Solutions, Guildford, United Kingdom) with a quadrature birdcage cardiac volume coil. After induction of inhalative anesthesia using isoflurane-oxygen (4–5%), animals were placed on a dedicated mouse sledge and MR-compatible ECG electrodes were attached to the paws. Anesthesia was maintained with isoflurane-oxygen (1.5–2%) to adjust heart rate at 400–450 beats per minute. Images were acquired using respiratory and ECG-gated gradient-echo cine sequences in two-chamber long-axis, four-chamber long-axis and five to seven short-axis planes completely covering the LV. Relevant acquisition parameters included: 15 phases per cardiac cycle, repetition time (TR) 10 ms, echo time (TE) 3 ms, averages 4, field of view (FOV) 40 × 40 mm, pixel size 0.15 × 0.15 mm, slice thickness 1 mm. All animals underwent two CMR examinations with a four-week time interval between each study.

### Left ventricular volumetric and functional analysis

Volumetric analysis was performed offline using commercially available software CMR^42^ (Circle Cardiovascular Imaging Inc., Calgary, Canada). LV end-diastolic (LV EDV) and end-systolic (LV ESV) volumes were quantified using manual planimetry of the endocardial and epicardial surface from short-axis stack and LV stroke volume (LV SV), LV EF, myocardial mass and cardiac output were calculated.

### Feature tracking

**Fig. 1:**
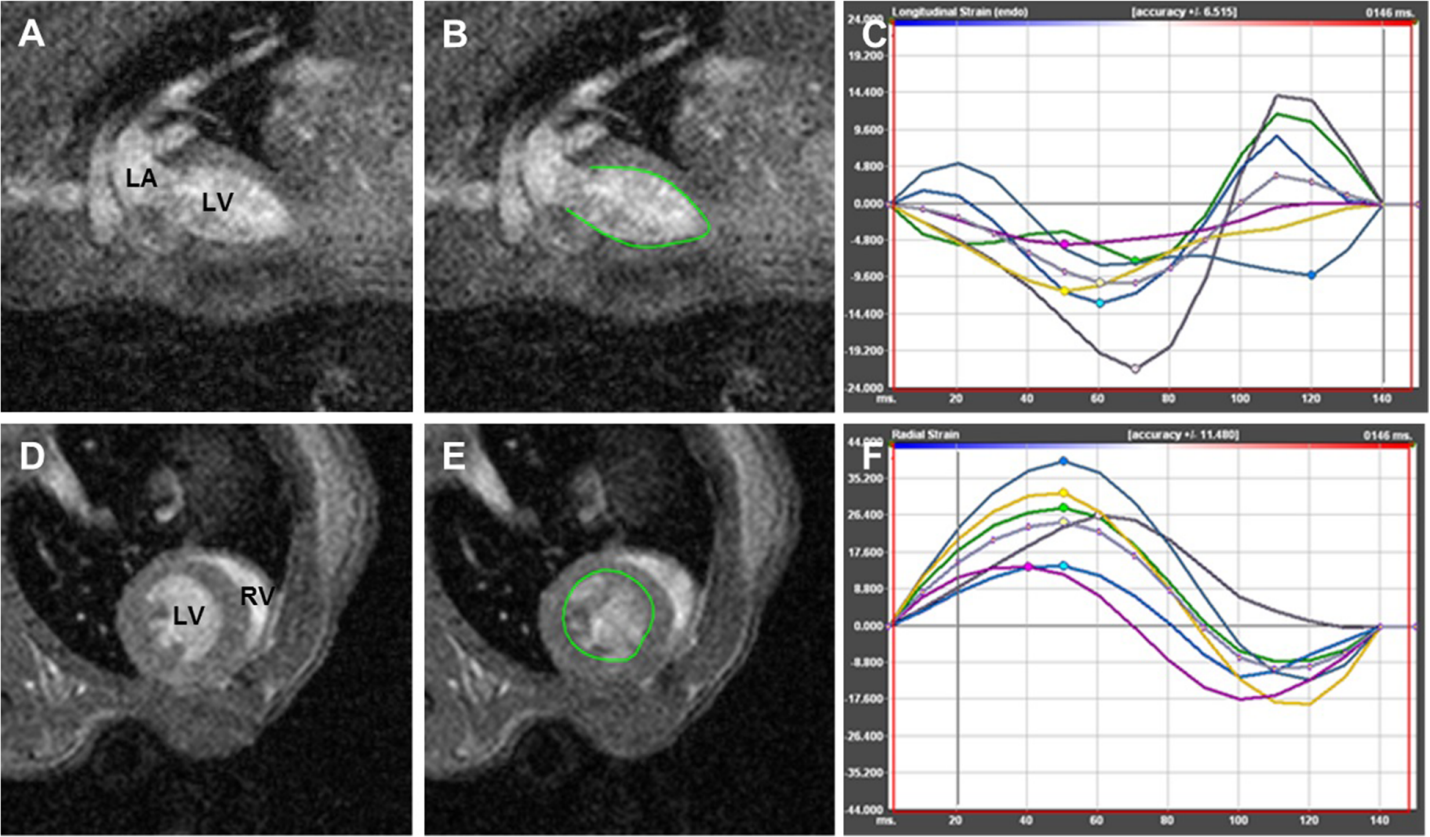
Cine CMR images without and with endocardial contouring and examples of CMR-FT myocardial strain curves in mouse. A frame from cine image of the LV two-chamber view depicting LV and LA (**a**). The same LV two-chamber cine image after application of tissue tracking algorithm (**b**) and example of LV longitudinal strain curves obtained from two-chamber cine image (**c**). Cine image of LV short-axis view at mid-ventricular level before (**d**) and after (**e**) automatic endocardial border detection. An example of LV radial strain curves derived from short-axis cine image (**f**). LV = left ventricle; LA = left atrium; CMR-FT = cardiovascular magnetic resonance feature tracking

The cine images were used to calculate myocardial strain and strain rate offline using dedicated software (TomTec Imaging Systems, 2D CPA, MR, Cardiac Performance Analysis, Unterschleissheim, Germany). Endocardial and epicardial contours were manually drawn in both long-axis and one mid-ventricular short-axis views at end-diastole for each mouse by two independent observers. After application of a tracking algorithm the software automatically identified endocardial borders throughout the cardiac cycle and computed mean segmental and global myocardial strain and strain rate parameters. All images were analyzed three times and derived measurements were averaged. LV global longitudinal strain and strain rate (Ell_LAX_ and SRll_LAX_) were calculated by averaging the strain curves of both two-chamber and four-chamber long-axis views (three measurements in two imaging planes of two scans in six mice resulted in 72 measurements) whereas global circumferential and radial strain (Ecc_SAX_ and Err_SAX_) and strain rate (SRcc_SAX_ and SRrr_SAX_) were derived using one mid-ventricular short-axis view containing both papillary muscles (three measurements in one imaging plane of two scans in six mice resulted in 36 measurements) (Fig. 1). To assess intra-observer agreement data analysis was repeated 4 weeks after initial assessment.

## Statistical analysis

### Reproducibility testing

Data was analyzed using Microsoft Excel and IBM SPSS Statistics version 23.0 software (SPSS Inc., Chicago, IL, USA) for Windows. Data are expressed as mean ± standard deviation (SD). The Shapiro-Wilk test was used to determine whether the data was normally distributed. Nonparametric variables were compared using the Wilcoxon test. A *p* value of <0.05 was considered statistically significant. Inter- and intra-observer reproducibility was quantified using intraclass correlation coefficient (ICC) and Bland-Altman analysis [27]. Agreement was considered excellent for ICC >0.74, good for ICC 0.60–0.74, fair for ICC 0.40–0.59, and poor for ICC <0.40 [28].

### Sample size calculation

Study sample size required to detect a relative 5, 8 and 10% change in strain with power of 90% and significance of 5% was calculated as follows [29]:

$$ n=f\left(\alpha, P\right)\bullet {\sigma}^2\bullet 2/{\delta}^2 $$

where *n* is the sample size, *α* the significance level, *P* the study power required and *f* the value of the factor for different values of *α* and *P* (*f* = 10.5 for *α* = 0.05 and *p* = 0.090), with *σ* the standard deviation of differences in measurements between two studies and *δ* the desired difference to be detected.

## Results

Demographic characteristics, volumetric and functional parameters of study population are summarized in Table 1. All studies were completed and image quality was sufficient to perform CMR-FT analysis. Table 2 demonstrates CMR-FT derived strain parameters obtained by two observers.

**Table 1:** Demographic, volumetric and functional characteristics of study subjects

Parameter	Value
Study population (n)	6
Male gender	6 (100%)
LV EDV (μl)	40.60 ± 10.15
LV ESV (μl)	18.02 ± 7.32
LV SV (μl)	22.57 ± 6.90
LV EF (%)	56.18 ± 10.70
Cardiac output (ml/min)	9.70 ± 3.27
LV Mass (mg)	58.87 ± 13.56

Results are reported as mean ± standard deviation. *LV* left ventricle / ventricular, *EDV* end-diastolic volume, *ESV* end-systolic volume, *SV* stroke volume, *EF* ejection fraction

**Table 2:** Comparison of CMR-FT derived strain parameters obtained by observers in six mice (72 measures for longitudinal and 36 measures for circumferential and radial strain respectively)

	Measurements obtained by two observers (inter-observer level)	
	First observer	Second observer
Ell_LAX_ (%)	−10.32 ± 5.47	−11.97 ± 6.36
Ecc_SAX_ (%)	−9.60 ± 3.68	−11.97 ± 5.97
Err_SAX_ (%)	12.59 ± 6.78	12.64 ± 7.23
	Measurements obtained by one observer (intra-observer level)	
	First measurement	Second measurement
Ell_LAX_ (%)	−10.32 ± 5.47	−11.57 ± 6.84
Ecc_SAX_ (%)	−9.60 ± 3.68	−8.48 ± 6.50
Err_SAX_ (%)	12.59 ± 6.78	10.32 ± 7.88

Results are expressed as mean ± standard deviation. *Ell*_*LAX*_ left ventricular long-axis longitudinal strain, *Ecc*_*SAX*_ left ventricular short-axis circumferential strain, *Err*_*SAX*_ left ventricular short-axis radial strain

### Inter-observer and intra-observer reproducibility

**Fig. 2:**
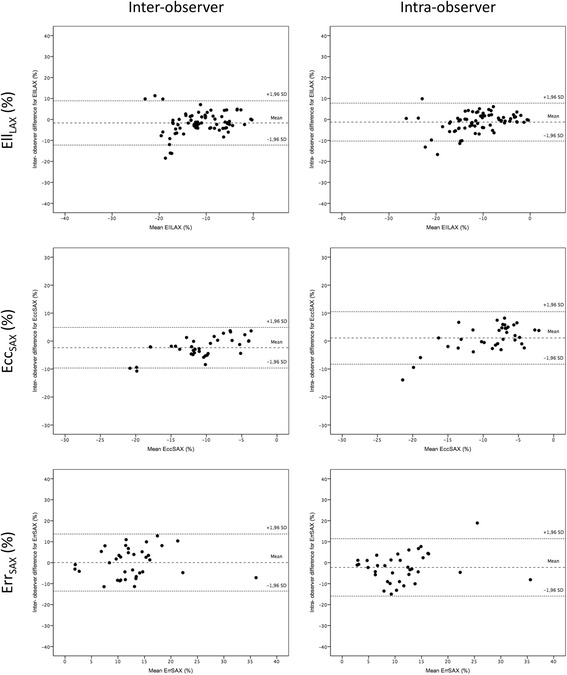
Bland-Altman plots with limits of agreement (1.96 standard deviations) demonstrates the inter-observer and intra-observer reproducibility of CMR-FT for LV strain parameters. The middle-dashed line is the mean of difference of measures. The upper and lower dotted lines are ±1.96 standard deviation. Ell_LAX_ = left ventricular long-axis longitudinal strain; Ecc_SAX_ = left ventricular short-axis circumferential strain; Err_SAX_ = left ventricular short-axis radial strain

The reproducibility for measurements was variable. Mean differences ± SD, limits of agreement and ICC for strain parameters are given in Table 3. There was excellent inter-observer reproducibility for Ecc_SAX_: ICC 0.79 (0.46–0.91) and Ell_LAX_: ICC 0.73 (0.56–0.83). In contrast, the level of intra-observer reproducibility was better for Ell_LAX_: ICC 0.83 (0.73–0.90) and lower for Ecc_SAX_: ICC 0.74 (0.49–0.87). The least reproducible measure for both observer levels was Err_SAX_: ICC 0.68 (0.37–0.84) and ICC 0.69 (0.41–0.84) for inter-observer and intra-observer level respectively. Bland-Altman plots demonstrate inter-observer and intra-observer reproducibility for left ventricular Ell_LAX_, Ecc_SAX_ and Err_SAX_ (Fig. 2).

**Table 3:** Inter-observer and intra-observer reproducibility for LV longitudinal, circumferential and radial strain

	Parameter	Mean difference ± SD	Limits of agreement	ICC (95% CI)
Inter-observer	Ell_LAX_ (%)	−1.65 ± 5.38	−12.2 to 8.90	0.73 (0.56 to 0.83)
	Ecc_SAX_ (%)	−2.37 ± 3.71	−9.64 to 4.89	0.79 (0.46 to 0.91)
	Err_SAX_ (%)	0.05 ± 6.94	−13.55 to 13.66	0.68 (0.37 to 0.84)
Intra-observer	Ell_LAX_ (%)	−1.25 ± 4.62	−10.30 to 7.79	0.83 (0.73 to 0.90)
	Ecc_SAX_ (%)	1.11 ± 4.79	−8.27 to 10.50	0.74 (0.49 to 0.87)
	Err_SAX_ (%)	−2.27 ± 6.98	−15.95 to 11.42	0.69 (0.41 to 0.84)

Results are reported as mean ± standard deviation. *ICC* intraclass correlation coefficient, *CI* confidence interval. Other abbreviations as in Table 2

### Sample size calculation

The change in reproducibility has an impact on sample size required to detect significant differences in strain parameters. Table 4 lists the required sample sizes for each strain measure. For example, to show a relative 10% change in Ecc_SAX_ in mice would require ten animals (not measures). In contrast, 85 mice are required to detect a 5% change in Err_SAX_ with CMR-FT (power of 90% and α error of 0.05).

**Table 4:** Sample size calculation for LV longitudinal, circumferential and radial strain to detect 5, 8 and 10% relative change with 90% power and α error of 0.05

Parameter	Mean difference ± SD	Sample size (n)		
		5%	8%	10%
Ell_LAX_ (%)	−0.98 ± 7.96	53	21	13
Ecc_SAX_ (%)	−3.46 ± 6.84	39	15	10
Err_SAX_ (%)	3.48 ± 10.06	85	33	21

Abbreviations as in Table 2

## Discussion

The current pilot study was designed to assess the inter-observer and intra-observer reproducibility of CMR-FT for the analysis of strain and strain rate in a small animal (mouse) model and to define the number of animals required for future studies. Our preliminary data analysis demonstrates very promising results:

- CMR-FT can be used for quantitative assessment of cardiac motion in small animals
- Good to excellent inter-observer and intra-observer reproducibility was found for LV circumferential and longitudinal strain, whereas radial strain is highly variable between repeated measurements
- Sample size calculation demonstrates relatively small sample size of animals (not measures) required to detect a 5, 8 and 10% change in strain parameters.

Small animal models play an important role in the understanding of cardiac mechanics in the various types of cardiovascular disease. There is growing evidence that advanced measures of myocardial deformation (strain or torsion) are better predictors of outcome compared with the routinely used ejection fraction or wall motion score index [30]. However, in the past the majority of animal experiments focused on changes in routine parameters as LV mass, volumes and/or function. The analysis of myocardial deformation offers quantitative assessment of early changes in LV diastolic function [31] and new findings in previously reported “negative” studies or studies with no obvious effect after therapy may improve our understanding of the underlying pathophysiology.

Previous studies have demonstrated the feasibility of two-dimensional (2D) speckle tracking echocardiography to assess myocardial motion in small animal models. Peng et al., evaluated the feasibility of circumferential and radial strain to detect LV dysfunction in a heart failure mouse models. They demonstrated that mice subjected to transverse aortic constriction experienced an immediate and sustained decrease in circumferential and radial strain [32]. The technical challenges with 2D echocardiography as limited imaging projections, small animal hearts and lower spatial resolution still limit its widespread use.

CMR-FT as a novel tissue tracking method became available in 2009 [33] and immediately evoked great interest in the CMR community. The CMR-FT algorithm tracks the endocardial and epicardial borders over time using conventional cine images. The advantages are that CMR-FT does not require acquisition of additional sequences and helps to protect animals from prolonged anesthesia. Furthermore, quantitative assessment of myocardial deformation can be performed retrospectively as cine images are a part of routine CMR study.

CMR-FT has been validated against the traditional CMR tagging techniques and showed excellent correlation between the two methods [34]. As opposed to speckle tracking echocardiography, only a small number of studies have been done using CMR tagging technique in small animal models [35, 36] and none using CMR-FT.

The temporal resolution of the tissue tracking technique is very important to meaningful interpretation of the results. If it is too low, an effect known as image de-correlation appears and the local patterns could become less comparable [4]. Under the conscious state the normal murine heart rate ranges from 500 to 700 beats per minute (bpm) [37]. During anesthesia, we were able to adjust mice heart rate at 400–450 bpm and achieve temporal resolution of 15 phases per cardiac cycle. There are no studies reporting minimal number of cardiac phases required to assess myocardial strain using CMR-FT. However, it should be mentioned that expected average temporal resolution in human studies is 25–35 phases per cardiac cycle (e.g., 30–40 ms at a heart rate of 60 bpm) [38]. Future studies assessing the impact of temporal resolution on the myocardial tracking accuracy of CMR-FT in animals and humans are necessary.

Higher reproducibility enables smaller changes to be detected with greater reliability. Comparative studies have reported that the most consistent parameters derived from CMR-FT are global circumferential and global longitudinal strain [33, 34]. Augustine et al., reported reasonable agreement between myocardial tagging and CMR-FT for global circumferential strain but not for longitudinal or radial strain. Inter-observer variability was acceptable for circumferential, but poor for radial strain [39]. Morton et al., evaluated inter-study reproducibility in healthy volunteers and found that global circumferential strain was the most reproducible measure [22]. Studies analyzing reproducibility of myocardial deformation parameters in small animal models are lacking. Haggery et al., conducted a study to evaluate the inter-observer and intra-observer variability in mice using stimulated echo CMR (DENSE) and reported high reproducibility for LV strains and torsion with better agreement of measurements at the inter-observer level. Myocardial strains were generally more reproducible than corresponding strain rates [40].

To improve reproducibility in our study, all measurements were derived three times. This strategy has been reported to increase the reproducibility of measurements in previous studies [10, 23]. Our study also demonstrates good to excellent inter-observer agreement for global circumferential and global longitudinal strain. In contrast, the reproducibility of longitudinal strain is better at the intra-observer level. However, the reproducibility of radial strain was weak, as expected and as reported in previous human studies [35].

The variability between measurements is an important factor for determining the ability of the technique to detect relevant differences between the individuals or significant change at follow-up examinations and becomes important not only in the context of experimental studies, but also in the clinical setting [29]. Clinical practice involves measuring of different parameters for a variety of purposes, such as diagnosis of the disease or prediction of future events. Measurements are almost always prone to various types of errors which make the measured value to differ from the true value. If these measurements are performed by different observers, differences may be due to bias of the observers [41].

In our study, inter-observer reproducibility and intra-observer reproducibility of the LV global circumferential strain were excellent while global longitudinal strain demonstrated good inter-observer and intra-observer agreement. The excellent reproducibility means that parameters could be used to detect differences between individuals or focus on intra-individual changes during follow-up studies.

Sample size calculation is an important aspect of study design and enables determination of how large the study sample should be. Too small sample size can miss the real effect, whereas too large sample size leads to unnecessary waste of time and resources (animals). Estimates of required sample size depend on the variability of the population – the greater the variability, the larger the required sample size. The most favored scientific method in experiments is calculation of sample size by power analysis. In the present pilot study, we estimated the variability of the difference because the standard deviation of a difference in measurement in an animal is lower than variability of the population. Therefore, the number of animals needed to test a hypothesis could be reduced because the effect of animal-to-animal variation on the measurement is eliminated [42]. In the light of the current discussion of improving animal welfare by methodological refinements to reduce suffering, ameliorated imaging techniques are an important tool to achieve it.

Reproducibility and sample size are affected by image quality and variability of measurements. We successfully performed measurements in all animals and to decrease variability of measurements used averaged results of repeated analyses. Nevertheless, our calculations could give the impression that sample sizes required to detect relative changes in strain parameters are too low (e.g. ten animals per group are needed to detect a relative 10% change in Ecc_SAX_ or 13 animals for Ell_LAX_). It is important to remember that the addition of a 25% dropout rate (proportion of eligible subjects who will not complete the study or provide only partial information) before planning a study will further increase the final sample size. Despite the small number of animals included in the study we could demonstrate that myocardial deformation parameters are highly reproducible. However, larger animal studies are necessary to confirm our preliminary findings.

### Limitations

Our study is limited due to the small number of animals and larger sample size may be required to detect more subtle differences. In addition, CMR-FT was performed on 2D and low temporal resolution (15 phases per cardiac cycle) cine images.

## Conclusions

Myocardial deformation parameters represent a new potential biomarker for the detection of early myocardial dysfunction. Cardiac mechanics parameters derived from conventional cine images using CMR-FT technique in small animal models are highly reproducible. The most reproducible measures are global circumferential and global longitudinal strain, whereas reproducibility of radial strain is weak. Relatively small study sample size could be sufficient to detect changes if parameter variability is low.

## Abbreviations

CMR: Cardiovascular magnetic resonance
Ecc_SAX_: Left ventricular short-axis circumferential strain
Ell_LAX_: Left ventricular long-axis longitudinal strain
Err_SAX_: Left ventricular short-axis radial strain
FT: Feature tracking
LV: Left ventricle / ventricular
SRcc_SAX_: Left ventricular short-axis circumferential strain rate
SRll_LAX_: Left ventricular long-axis longitudinal strain rate
SRrr_SAX_: Left ventricular short-axis radial strain rate

## References

[1] St John Sutton M, Pfeffer MA, Moye L, Plappert T, Rouleau JL, Lamas G, Rouleau J, Parker JO, Arnold MO, Sussex B, Braunwald E (1997). Cardiovascular death and left ventricular remodeling two years after myocardial infarction: baseline predictors and impact of long-term use of captopril: information from the survival and ventricular enlargement (SAVE) trial. Circulation.

[2] Cikes M, Solomon SD (2016). Beyond ejection fraction: an integrative approach for assessment of cardiac structure and function in heart failure. Eur Heart J.

[3] Ibrahim E-SH (2011). Myocardial tagging by cardiovascular magnetic resonance: evolution of techniques – pulse sequences, analysis algorithms, and applications. J Cardiovasc Magn Reson.

[4] Pedrizzetti G, Claus P, Kilner PJ, Nagel E (2016). Principles of cardiovascular magnetic resonance feature tracking and echocardiographic speckle tracking for informed clinical use. J Cardiovasc Magn Reson.

[5] Claus P, Omar AM, Pedrizzetti G, Sengupta PP, Nagel E (2015). Tissue tracking technology for assessing cardiac mechanics: principles, normal values, and clinical applications. JACC Cardiovasc Imaging..

[6] Zerhouni EA, Parish DM, Rogers WJ, Yang A, Shapiro EP (1988). Human heart: tagging with MR imaging – a method for noninvasive assessment of myocardial motion. Radiology.

[7] Axel L, Dougherty L (1989). MR imaging of motion with spatial modulation of magnetization. Radiology.

[8] Wang J, Khoury DS, Thohan V, Torre-Amione G, Nagueh SF (2007). Global diastolic strain rate for the assessment of left ventricular relaxation and filling pressures. Circulation.

[9] Schneeweis C, Lapinskas T, Schnackenburg B, Berger A, Hucko T, Kelle S, Fleck E, Gebker R (2014). Comparison of myocardial tagging and feature tracking in patients with severe aortic stenosis. J Heart Valve Dis.

[10] Kowallick JT, Kutty S, Edelmann F, Chiribiri A, Villa A, Steinmetz M, Sohns JM, Staab W, Bettencourt N, Unterberg-Buchwald C, Hasenfuß G, Lotz J, Schuster A (2014). Quantification of left atrial strain and strain rate using cardiovascular magnetic resonance myocardial feature tracking: a feasibility study. J Cardiovasc Magn Reson.

[11] Kempny A, Fernández-Jiménez R, Orwat S, Schuler P, Bunck AC, Maintz D, Baumgartner H, Diller GP (2012). Quantification of biventricular myocardial function using cardiac magnetic resonance feature tracking, endocardial border delineation and echocardiographic speckle tracking in patients with repaired tetralogy of Fallot and healthy controls. J Cardiovasc Magn Reson.

[12] Schneeweis C, Qiu J, Schnackenburg B, Berger A, Kelle S, Fleck E, Gebker R (2014). Value of additional strain analysis with feature tracking in dobutamine stress cardiovascular magnetic resonance for detecting coronary artery disease. J Cardiovasc Magn Reson.

[13] Meyer CG, Frick M, Lotfi S, Altiok E, Koos R, Kirschfink A, Lehrke M, Autschbach R, Hoffmann R (2014). Regional left ventricular function after transapical vs transfemoral transcatheter aortic valve implantation analysed by cardiac magnetic resonance feature tracking. Eur Heart J Cardiovasc Imaging..

[14] Morais P, Marchi A, Bogaert JA, Dresselaers T, Heyde B, D’hooge J, Bogaert J (2017). Cardiovascular magnetic resonance myocardial feature tracking using a non-rigid, elastic image registration algorithm: assessment of variability in a real-life clinical setting. J Cardiovasc Magn Reson.

[15] Kraitchman DL, Sampath S, Castillo E, Derbyshire JA, Boston RC, Bluemke DA, Gerber BL, Prince JL, Osman NF (2003). Quantitative ischemia detection during cardiac magnetic resonance stress testing by use of FastHARP. Circulation.

[16] Moody WE, Taylor RJ, Edwards NC, Chue CD, Umar F, Taylor TJ, Ferro CJ, Young AA, Townend JN, Leyva F, Steeds RP (2015). Comparison of magnetic resonance feature tracking for systolic and diastolic strain and strain rate calculation with spatial modulation of magnetization imaging analysis. J Magn Reson Imaging.

[17] Nahum J, Bensaid A, Dussault C, Macron L, Clémence D, Bouhemad B, Monin JL, Rande JL, Gueret P, Lim P (2010). Impact of longitudinal myocardial deformation on the prognosis of chronic heart failure patients. Circ Cardiovasc Imaging..

[18] Smith BM, Dorfman AL, Yu S, Russell MW, Agarwal PP, Ghadimi Mahani M, Lu JC (2014). Relation of strain by feature tracking and clinical outcome in children, adolescents, and young adults with hypertrophic cardiomyopathy. Am J Cardiol.

[19] Buss SJ, Breuninger K, Lehrke S, Voss A, Galuschky C, Lossnitzer D, Andre F, Ehlermann P, Franke J, Taeger T, Frankenstein L, Steen H, Meder B, Giannitsis E, Katus HA, Korosoglou G (2015). Assessment of myocardial deformation with cardiac magnetic resonance strain imaging improves risk stratification in patients with dilated cardiomyopathy. Eur Heart J Cardiovasc Imaging.

[20] Mahfoud F, Urban D, Teller D, Linz D, Stawowy P, Hassel JH, Fries P, Dreysse S, Wellnhofer E, Schneider G, Buecker A, Schneeweis C, Doltra A, Schlaich MP, Esler MD, Fleck E, Böhm M, Kelle S (2014). Effect of renal denervation on left ventricular mass and function in patients with resistant hypertension: data from a multi-centre cardiovascular magnetic resonance imaging trial. Eur Heart J.

[21] Bernard A, Donal E, Leclercq C, Schnell F, Fournet M, Reynaud A, Thebault C, Mabo P, Daubert JC, Hernandez A (2015). Impact of cardiac resynchronization therapy on left ventricular mechanics: understanding the response through a new quantitative approach based on longitudinal strain integrals. J Am Soc Echocardiogr.

[22] Morton G, Schuster A, Jogiya R, Kutty S, Beerbaum P, Nagel E (2012). Inter-study reproducibility of cardiovascular magnetic resonance myocardial feature tracking. J Cardiovasc Magn Reson.

[23] Schuster A, Paul M, Bettencourt N, Hussain ST, Morton G, Kutty S, Bigalke B, Chiribiri A, Perera D, Nagel E, Beerbaum P (2015). Myocardial feature tracking reduces observer-dependance in low-dose dobutamine stress cardiovascular magnetic resonance. PLoS One.

[24] Lapinskas T, Bučius P, Urbonaitė L, Stabinskaitė A, Valuckienė Ž, Jankauskaitė L, Benetis R, Žaliūnas R (2017). Left atrial mechanics in patients with acute STEMI and secondary mitral regurgitation: a prospective pilot CMR feature tracking study. Medicina (Kaunas).

[25] Kowallick JT, Morton G, Lamata P, Jogiya R, Kutty S, Lotz J, Hasenfuß G, Nagel E, Chiribiri A, Schuster A (2016). Inter-study reproducibility of left ventricular torsion and torsion rate quantification using MR myocardial feature tracking. J Magn Reson Imaging.

[26] Schuster A, Morton G, Hussain ST, Jogiya R, Kutty S, Asrress KN, Makowski MR, Bigalke B, Perera D, Beerbaum P, Nagel E (2013). The intra-observer reproducibility of cardiovascular magnetic resonance myocardial feature tracking strain assessment is independent of field strength. Eur J Radiol.

[27] Bland JM, Altman DG (1996). Statistical methods for assessing agreement between two methods of clinical measurement. Lancet.

[28] Oppo K, Leen E, Angerson WJ, Cooke TG, McArdle CS (1998). Doppler perfusion index: an interobserver and intraobserver reproducibility study. Radiology.

[29] Grothues F, Smith GC, Moon JC, Bellenger NG, Collins P, Klein HU, Pennell DJ (2002). Comparison of interstudy reproducibility of cardiovascular magnetic resonance with two-dimensional echocardiography in normal subjects and in patients with heart failure or left ventricular hypertrophy. Am J Cardiol.

[30] Stanton T, Leano R, Marwick TH (2009). Prediction of all-cause mortality from global longitudinal speckle strain: comparison with ejection fraction and wall motion scoring. Circ Cardiovasc Imaging..

[31] Flachskampf FA, Biering-Sørensen T, Solomon SD, Duvernoy O, Bjener T, Smiseth OA (2015). Cardiac imaging to evaluate left ventricular diastolic function. JACC Cardiovasc Imaging..

[32] Peng Y, Popovic ZB, Sopko N, Drinko J, Zhang Z, Thomas JD, Penn MS (2009). Speckle tracking echocardiography in the assessment of mouse models of cardiac dysfunction. Am J Physiol Heart Circ Physiol.

[33] Maret E, Todt T, Brudin L, Nylander E, Swahn E, Ohlsson JL, Engvall JE (2009). Functional measurements based on feature tracking of cine magnetic resonance images identify left ventricular segments with myocardial scar. Cardiovasc Ultrasound.

[34] Hor KN, Gottliebson WM, Carson C, Wash E, Cnota J, Fleck R, Wansapura J, Klimeczek P, Al-Khalidi HR, Chung ES, Benson DW, Mazur W (2010). Comparison of magnetic resonance feature tracking for strain calculation with harmonic phase imaging analysis. JACC Cardiovasc Imaging.

[35] Zhong J, Liu W, Yu X (2008). Characterization of three-dimensional myocardial deformation in the mouse heart: an MR tagging study. J Magn Reson Imaging.

[36] Kramer SP, Powell DK, Haggerty CM, Binkley CM, Mattingly AC, Cassis LA, Epstein FH, Fornwalt BK (2013). Obesity reduces left ventricular strains, torsion, and synchrony in mouse models: a cine displacement encoding with stimulated echoes (DENSE) cardiovascular magnetic resonance study. J Cardiovasc Magn Reson.

[37] Ho D, Zhao X, Gao S, Hong C, Vatner DE, Vatner SF (2011). Heart rate and electrocardiography monitoring in mice. Curr Protoc Mouse Biol.

[38] Schuster A, Hor KN, Kowallick JT, Beerbaum P, Kutty S (2016). Cardiovascular magnetic resonance myocardial feature tracking: concepts and clinical applications. Circ Cardiovasc Imaging.

[39] Augustine D, Lewandowski AJ, Lazdam M, Rai A, Francis J, Myerson S, Noble A, Becher H, Neubauer S, Petersen SE, Leeson P (2013). Global and regional left ventricular myocardial deformation measures by magnetic resonance feature tracking in healthy volunteers: comparison with tagging and relevance of gender. J Cardiovasc Magn Reson.

[40] Haggerty CM, Kramer SP, Binkley CM, Powell DK, Mattingly AC, Charnigo R, Epstein FH, Fornwalt BK (2013). Reproducibility of cine displacement encoding with stimulated echoes (DENSE) cardiovascular magnetic resonance for measuring left ventricular strains, torsion, and synchrony in mice. J Cardiovasc Magn Reson.

[41] Bartlett JW, Frost C (2008). Reliability, repeatability and reproducibility: analysis of measurement errors in continuous variables. Ultrasound Obstet Gynecol.

[42] Dell RB, Holleran S, Ramakrishnan R (2002). Sample size determination. ILAR J.

